# Diethyl [(3-cyano-1-phenyl­sulfonyl-1*H*-indol-2-yl)meth­yl]phospho­nate

**DOI:** 10.1107/S1600536811008038

**Published:** 2011-03-09

**Authors:** S. Karthikeyan, K. Sethusankar, Ganesan Gobi Rajeshwaran, Arasambattu K. Mohanakrishnan, D. Velmurugan

**Affiliations:** a Department of Physics, RKM Vivekananda College (Autonomous), Chennai 600 004, India; b Department of Organic Chemistry, University of Madras, Maraimalai Campus, Chennai 600 025, India; c Centre of Advanced Study in Crystallography and Biophysics, University of Madras, Maraimalai Campus, Chennai 600 025, India

## Abstract

In the title compound, C_20_H_21_N_2_O_5_PS, the indole ring is essentially planar, with a maximum deviation of −0.0083 (18) Å. The methyl C atom of the methyl­phospho­nate group and the S atom lie 0.104 (2) and −0.2158 (6) Å, respectively, from the indole mean plane. The sulfonyl-bound phenyl ring is almost perpendicular to the indole ring system, with a dihedral angle of 82.30 (8)°. The ethyl side chains are disordered over two sets of sites, with occupancy factors of 0.737 (5)/0.263 (5) and 0.529 (11)/0.471 (11). In the crystal, mol­ecules are linked into centrosymmetric dimers *via* C—H⋯O hydrogen bonds, resulting in an *R*_2_^2^(18) graph-set motif. The crystal structure is further stabilized by C—H⋯π inter­actions.

## Related literature

For applications of indole derivatives, see: Stevenson *et al.* (2000 ▶); Ho *et al.* (1986 ▶); Rajeswaran *et al.* (1999 ▶). For comparison of mol­ecular dimensions, see: Bassindale (1984 ▶); Sethu Sankar *et al.* (2002 ▶); Allen (1981 ▶). For graph-set motif notations, see: Bernstein *et al.* (1995 ▶).
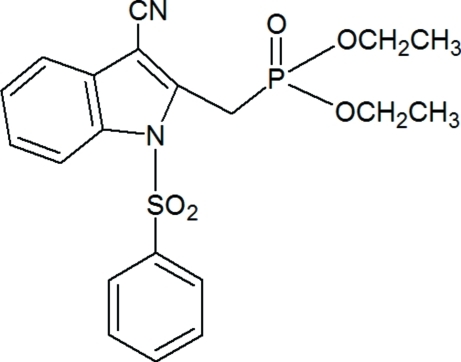


## Experimental

### 

#### Crystal data

C_20_H_21_N_2_O_5_PS
*M* *_r_* = 432.42
Triclinic, 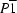

*a* = 9.198 (5) Å
*b* = 11.229 (5) Å
*c* = 11.992 (5) Å
α = 65.569 (5)°
β = 72.950 (5)°
γ = 72.204 (5)°
*V* = 1053.7 (9) Å^3^
*Z* = 2
Mo *K*α radiation
μ = 0.26 mm^−1^
*T* = 293 K
0.23 × 0.20 × 0.20 mm

#### Data collection

Bruker SMART APEXII area-detector diffractometer
19331 measured reflections
5172 independent reflections
4201 reflections with *I* > 2σ(*I*)
*R*_int_ = 0.025

#### Refinement

*R*[*F* ^2^ > 2σ(*F* ^2^)] = 0.042
*wR*(*F* ^2^) = 0.126
*S* = 1.02
5172 reflections
284 parameters
10 restraints
H-atom parameters constrained
Δρ_max_ = 0.28 e Å^−3^
Δρ_min_ = −0.33 e Å^−3^

### 

Data collection: *APEX2* (Bruker, 2004 ▶); cell refinement: *SAINT* (Bruker, 2004 ▶); data reduction: *SAINT*; program(s) used to solve structure: *SHELXS97* (Sheldrick, 2008 ▶); program(s) used to refine structure: *SHELXL97* (Sheldrick, 2008 ▶); molecular graphics: *ORTEP-3* (Farrugia, 1997 ▶); software used to prepare material for publication: *SHELXL97* and *PLATON* (Spek, 2009 ▶).

## Supplementary Material

Crystal structure: contains datablocks global, I. DOI: 10.1107/S1600536811008038/pv2393sup1.cif

Structure factors: contains datablocks I. DOI: 10.1107/S1600536811008038/pv2393Isup2.hkl

Additional supplementary materials:  crystallographic information; 3D view; checkCIF report

## Figures and Tables

**Table 1:** Hydrogen-bond geometry (Å, °) *Cg*1 is the centroid of the C9/C10/C11/C12/C13/C14 ring and *Cg*2 is the centroid of the C1/C2/C3/C4/C5/C6 ring.

*D*—H⋯*A*	*D*—H	H⋯*A*	*D*⋯*A*	*D*—H⋯*A*
C10—H10⋯O5^i^	0.93	2.35	3.229 (3)	157
C5—H5⋯*Cg*1^ii^	0.93	2.63	3.501 (3)	157
C18—H18*A*⋯*Cg*2^iii^	0.96	2.99	3.874 (8)	154

Symmetry codes: (i) 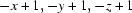; (ii) 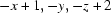; (iii) 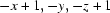.

**Fig. 1.**
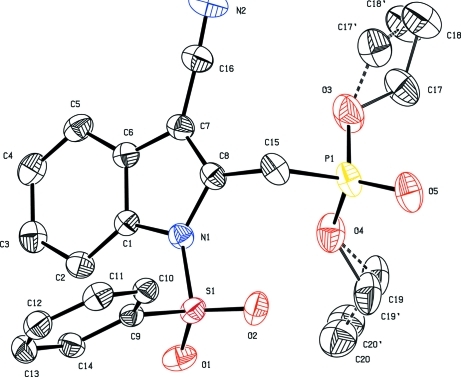
The molecular structure of the title compound showing 30% probability displacement ellipsoids. Ethyl groups attached on O4 are disordered.

**Fig. 2.**
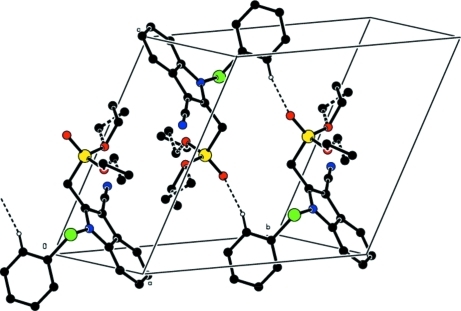
A unit cell packing of the crystal structure of the title compound, showing H-bonds.

## supplementary crystallographic information

### Comment

The indole ring system is present in many natural products. Indole derivaties are used as bioactive drugs (Stevenson, *et al.*, 2000) and they exhibit anti-allergic, central nervous system depressant and muscle relaxant properties (Ho, *et al.*, 1986). Indoles also have been proved to display high aldose reductase inhibitory activity (Rajeswaran, *et al.*, 1999).

In the title compound (Fig. 1), ethyl moieties of diethyl phosphonate are disordered over two sites with occupancy factors 0.737 (5), 0.263 (5) and 0.529 (11), 0.471 (11). The indole ring is essentially planar with a maximum deviation -0.0083 (18) Å for the atom C6. The deviation of atoms C15 and S1 from the indole mean plane is 0.1041 (22) and -0.2158 (6) Å, respectively. The sulfonyl bound phenyl ring is almost perpendicular to the indole ring system, with a dihedral angle of 82.30 (8)°. The atom P1 has a distorted tetrahedral configuration. The widening of angle O3—P1—O5 [115.78 (10)°] and narrowing of angle O4—P1—C15 [104.97 (9)°] from the ideal tetrahedral value are attributed to the Thrope-Ingold effect (Bassindale, 1984).

In the benzene ring of the indole ring system, the endocyclic angles at C2 and C5 are contracted to 117.49 (17) and 118.56 (17)° respectively, while those at C1, C3 and C4 are expanded to 121.35 (15)°, 121.69 (18) and 120.69 (17)°, respectively. This would appear to be a real effect caused by the fusion of the smaller pyrrole ring to the six-membered benzene ring and the strain is taken up by the angular distortion rather than by bond-length distortions (Allen, 1981; Sethu Sankar *et al.*, 2002).

In the crystal, molecules are linked into centrosymmetric dimers *via* C–H···O hydrogen bonds resulting in a *R*^2^_2_(18) graphset motif (Bernstein *et al.*, 1995). The crystal structure is further stabilized by C–H···π interactions, where *Cg*(1) is the centroid of C9/C10/C11/C12/13/C14 ring and *Cg*(2) is the centroid of C1/C2/C3/C4/C5/C6 ring.

### Experimental

To a solution of 2-(bromomethyl)-1-phenylsulfonyl-indole-3-carbonitrile (1 mmol) and triethylphosphite (1.2 mmol) in dry dichloromethane (10 ml) at room temperature, ZnBr_2_ (0.2 mmol) was added and allowed to stir for 2 h under N_2_.  After consumption of the bromo compound (monitored by TLC) volatile components were removed under vacuo. The residual mass was poured over crushed ice (200 g) containing conc. HCl (5 ml). The precipitated solid was filtered, washed with water and dried to give crude phosphonate ester. The crude product was purified by flash column chromatography to provide the title compound which was recrystalized from a mixture of 50% ethylacetate in pure hexane.

### Refinement

All the hydrogen atoms weree fixed geometrically and allowed to ride on their parent atoms with C—H distance in the range 0.93Å to 0.97Å and with *U*_iso_(H) = 1.5*U*_eq_(C) for CH_3_ groups and *U*_iso_(H) = 1.2*U*_eq_(C) for all the other H-atoms.

### Crystal data

**Table:**

C_20_H_21_N_2_O_5_PS	*Z* = 2
*M**_r_* = 432.42	*F*(000) = 452
Triclinic, *P*1	*D*_x_ = 1.363 Mg m^−^^3^
Hall symbol: -P 1	Mo *K*α radiation, λ = 0.71073 Å
*a* = 9.198 (5) Å	Cell parameters from 5172 reflections
*b* = 11.229 (5) Å	θ = 1.0–28.2°
*c* = 11.992 (5) Å	µ = 0.26 mm^−^^1^
α = 65.569 (5)°	*T* = 293 K
β = 72.950 (5)°	Block, colourless
γ = 72.204 (5)°	0.23 × 0.20 × 0.20 mm
*V* = 1053.7 (9) Å^3^	

### Data collection

**Table:**

Bruker SMART APEXII area-detector diffractometer	4201 reflections with *I* > 2σ(*I*)
Radiation source: fine-focus sealed tube	*R*_int_ = 0.025
graphite	θ_max_ = 28.2°, θ_min_ = 1.9°
ω scans	*h* = −12→12
19331 measured reflections	*k* = −14→14
5172 independent reflections	*l* = −15→15

### Refinement

**Table:**

Refinement on *F*^2^	Primary atom site location: structure-invariant direct methods
Least-squares matrix: full	Secondary atom site location: difference Fourier map
*R*[*F*^2^ > 2σ(*F*^2^)] = 0.042	Hydrogen site location: inferred from neighbouring sites
*wR*(*F*^2^) = 0.126	H-atom parameters constrained
*S* = 1.02	*w* = 1/[σ^2^(*F*_o_^2^) + (0.0647*P*)^2^ + 0.2952*P*] where *P* = (*F*_o_^2^ + 2*F*_c_^2^)/3
5172 reflections	(Δ/σ)_max_ < 0.001
284 parameters	Δρ_max_ = 0.28 e Å^−^^3^
10 restraints	Δρ_min_ = −0.33 e Å^−^^3^

### Special details

**Table:**

Geometry. All e.s.d.'s (except the e.s.d. in the dihedral angle between two l.s. planes) are estimated using the full covariance matrix. The cell e.s.d.'s are taken into account individually in the estimation of e.s.d.'s in distances, angles and torsion angles; correlations between e.s.d.'s in cell parameters are only used when they are defined by crystal symmetry. An approximate (isotropic) treatment of cell e.s.d.'s is used for estimating e.s.d.'s involving l.s. planes.
Refinement. Refinement of *F*^2^ against ALL reflections. The weighted *R*-factor *wR* and goodness of fit *S* are based on *F*^2^, conventional *R*-factors *R* are based on *F*, with *F* set to zero for negative *F*^2^. The threshold expression of *F*^2^ > σ(*F*^2^) is used only for calculating *R*-factors(gt) *etc*. and is not relevant to the choice of reflections for refinement. *R*-factors based on *F*^2^ are statistically about twice as large as those based on *F*, and *R*- factors based on ALL data will be even larger.

### Fractional atomic coordinates and isotropic or equivalent isotropic displacement parameters (Å^2^)

**Table:**

	*x*	*y*	*z*	*U*_iso_*/*U*_eq_	Occ. (<1)
C1	0.29990 (18)	0.07457 (15)	0.89632 (14)	0.0423 (3)	
C2	0.1831 (2)	0.03001 (18)	0.99695 (17)	0.0517 (4)	
H2	0.0877	0.0869	1.0109	0.062*	
C3	0.2138 (2)	−0.10164 (19)	1.07533 (18)	0.0581 (4)	
H3	0.1379	−0.1337	1.1438	0.070*	
C4	0.3557 (2)	−0.18786 (19)	1.05461 (19)	0.0600 (5)	
H4	0.3727	−0.2763	1.1089	0.072*	
C5	0.4706 (2)	−0.14371 (18)	0.95485 (18)	0.0556 (4)	
H5	0.5651	−0.2015	0.9406	0.067*	
C6	0.44273 (18)	−0.01035 (16)	0.87526 (15)	0.0446 (3)	
C7	0.53430 (19)	0.06663 (18)	0.76474 (16)	0.0483 (4)	
C8	0.45078 (19)	0.19408 (17)	0.72042 (15)	0.0460 (3)	
C9	0.19475 (17)	0.38218 (15)	0.91760 (14)	0.0411 (3)	
C10	0.29566 (19)	0.46655 (16)	0.88290 (16)	0.0475 (4)	
H10	0.3488	0.4974	0.8010	0.057*	
C11	0.3155 (2)	0.50389 (19)	0.97293 (19)	0.0570 (4)	
H11	0.3817	0.5614	0.9512	0.068*	
C12	0.2382 (2)	0.4566 (2)	1.09435 (19)	0.0602 (5)	
H12	0.2535	0.4816	1.1543	0.072*	
C13	0.1383 (2)	0.3728 (2)	1.12775 (17)	0.0588 (4)	
H13	0.0868	0.3410	1.2101	0.071*	
C14	0.11430 (19)	0.33556 (17)	1.03911 (16)	0.0497 (4)	
H14	0.0454	0.2803	1.0607	0.060*	
C15	0.4982 (2)	0.30307 (19)	0.60283 (17)	0.0569 (4)	
H15A	0.6102	0.2928	0.5868	0.068*	
H15B	0.4507	0.3885	0.6136	0.068*	
C16	0.6889 (2)	0.0160 (2)	0.7105 (2)	0.0619 (5)	
N1	0.30429 (16)	0.20145 (13)	0.79986 (12)	0.0447 (3)	
N2	0.8120 (2)	−0.0289 (2)	0.6719 (2)	0.0908 (6)	
O1	0.02186 (14)	0.29595 (14)	0.84344 (14)	0.0623 (3)	
O2	0.19567 (18)	0.44146 (13)	0.68448 (12)	0.0643 (4)	
O3	0.5622 (2)	0.17770 (18)	0.44876 (15)	0.0926 (6)	
O4	0.2857 (2)	0.26002 (16)	0.52126 (14)	0.0762 (4)	
O5	0.4461 (2)	0.42928 (16)	0.36258 (14)	0.0822 (5)	
P1	0.44364 (7)	0.30451 (5)	0.46950 (4)	0.06049 (16)	
S1	0.16477 (5)	0.33911 (4)	0.80224 (4)	0.04691 (13)	
C17	0.5975 (4)	0.1469 (4)	0.3410 (3)	0.0885 (12)	0.737 (5)
H17A	0.5380	0.2176	0.2809	0.106*	0.737 (5)
H17B	0.5646	0.0648	0.3618	0.106*	0.737 (5)
C18	0.7638 (6)	0.1305 (6)	0.2820 (5)	0.1064 (15)	0.737 (5)
H18A	0.7789	0.1097	0.2089	0.160*	0.737 (5)
H18B	0.8237	0.0591	0.3398	0.160*	0.737 (5)
H18C	0.7971	0.2122	0.2588	0.160*	0.737 (5)
C17'	0.7241 (10)	0.1678 (10)	0.3780 (9)	0.086 (3)	0.263 (5)
H17C	0.7981	0.1300	0.4334	0.103*	0.263 (5)
H17D	0.7422	0.2546	0.3186	0.103*	0.263 (5)
C18'	0.734 (2)	0.0762 (17)	0.3133 (16)	0.1064 (15)	0.263 (5)
H18D	0.8365	0.0629	0.2635	0.160*	0.263 (5)
H18E	0.6585	0.1152	0.2605	0.160*	0.263 (5)
H18F	0.7145	−0.0083	0.3741	0.160*	0.263 (5)
C20	0.010 (2)	0.284 (3)	0.543 (3)	0.097 (3)	0.471 (11)
H20A	−0.0705	0.3064	0.4969	0.146*	0.471 (11)
H20B	−0.0126	0.3450	0.5855	0.146*	0.471 (11)
H20C	0.0135	0.1942	0.6030	0.146*	0.471 (11)
C19	0.1657 (12)	0.2917 (14)	0.4538 (10)	0.0901 (19)	0.471 (11)
H19A	0.1887	0.2294	0.4109	0.108*	0.471 (11)
H19B	0.1612	0.3812	0.3916	0.108*	0.471 (11)
C19'	0.1460 (10)	0.3402 (10)	0.4735 (9)	0.0901 (19)	0.529 (11)
H19C	0.1681	0.3736	0.3830	0.108*	0.529 (11)
H19D	0.1046	0.4160	0.5013	0.108*	0.529 (11)
C20'	0.031 (2)	0.250 (2)	0.524 (3)	0.097 (3)	0.529 (11)
H20D	−0.0640	0.2992	0.4932	0.146*	0.529 (11)
H20E	0.0086	0.2188	0.6134	0.146*	0.529 (11)
H20F	0.0737	0.1748	0.4968	0.146*	0.529 (11)

### Atomic displacement parameters (Å^2^)

**Table:**

	*U*^11^	*U*^22^	*U*^33^	*U*^12^	*U*^13^	*U*^23^
C1	0.0458 (8)	0.0407 (7)	0.0454 (8)	−0.0100 (6)	−0.0116 (6)	−0.0177 (6)
C2	0.0478 (9)	0.0529 (9)	0.0565 (10)	−0.0140 (7)	−0.0071 (7)	−0.0213 (8)
C3	0.0648 (11)	0.0585 (10)	0.0547 (10)	−0.0280 (9)	−0.0091 (8)	−0.0148 (8)
C4	0.0738 (12)	0.0454 (9)	0.0643 (11)	−0.0148 (8)	−0.0257 (9)	−0.0130 (8)
C5	0.0589 (10)	0.0480 (9)	0.0654 (11)	−0.0010 (8)	−0.0247 (8)	−0.0242 (8)
C6	0.0459 (8)	0.0472 (8)	0.0501 (8)	−0.0088 (6)	−0.0133 (6)	−0.0241 (7)
C7	0.0452 (8)	0.0564 (9)	0.0525 (9)	−0.0110 (7)	−0.0076 (7)	−0.0293 (8)
C8	0.0500 (8)	0.0523 (9)	0.0453 (8)	−0.0169 (7)	−0.0039 (6)	−0.0258 (7)
C9	0.0399 (7)	0.0385 (7)	0.0432 (8)	−0.0019 (6)	−0.0098 (6)	−0.0163 (6)
C10	0.0467 (8)	0.0443 (8)	0.0478 (8)	−0.0078 (6)	−0.0086 (7)	−0.0144 (7)
C11	0.0567 (10)	0.0517 (9)	0.0700 (12)	−0.0097 (8)	−0.0208 (9)	−0.0244 (9)
C12	0.0672 (11)	0.0608 (11)	0.0595 (11)	0.0017 (9)	−0.0225 (9)	−0.0319 (9)
C13	0.0642 (11)	0.0598 (10)	0.0447 (9)	−0.0009 (8)	−0.0066 (8)	−0.0223 (8)
C14	0.0470 (8)	0.0458 (8)	0.0501 (9)	−0.0070 (7)	−0.0026 (7)	−0.0177 (7)
C15	0.0676 (11)	0.0574 (10)	0.0538 (10)	−0.0297 (9)	0.0026 (8)	−0.0256 (8)
C16	0.0532 (10)	0.0684 (12)	0.0672 (12)	−0.0110 (9)	−0.0046 (8)	−0.0332 (10)
N1	0.0486 (7)	0.0414 (7)	0.0446 (7)	−0.0091 (5)	−0.0059 (5)	−0.0181 (6)
N2	0.0581 (11)	0.1031 (16)	0.1028 (16)	−0.0033 (10)	0.0044 (10)	−0.0518 (13)
O1	0.0479 (7)	0.0622 (8)	0.0876 (10)	−0.0041 (6)	−0.0241 (6)	−0.0349 (7)
O2	0.0906 (10)	0.0512 (7)	0.0476 (7)	−0.0059 (7)	−0.0263 (6)	−0.0120 (6)
O3	0.1255 (15)	0.0875 (12)	0.0632 (9)	−0.0091 (10)	−0.0062 (9)	−0.0427 (9)
O4	0.0941 (11)	0.0808 (10)	0.0583 (8)	−0.0476 (9)	−0.0170 (7)	−0.0070 (7)
O5	0.1092 (13)	0.0757 (10)	0.0518 (8)	−0.0455 (9)	0.0009 (8)	−0.0060 (7)
P1	0.0823 (4)	0.0591 (3)	0.0423 (2)	−0.0321 (3)	0.0031 (2)	−0.0183 (2)
S1	0.0508 (2)	0.0430 (2)	0.0495 (2)	−0.00350 (16)	−0.01729 (17)	−0.01830 (17)
C17	0.103 (3)	0.107 (3)	0.077 (2)	−0.028 (2)	0.0013 (18)	−0.062 (2)
C18	0.099 (3)	0.115 (5)	0.101 (3)	−0.021 (3)	0.007 (2)	−0.053 (4)
C17'	0.110 (8)	0.073 (6)	0.079 (6)	−0.026 (5)	−0.010 (5)	−0.034 (5)
C18'	0.099 (3)	0.115 (5)	0.101 (3)	−0.021 (3)	0.007 (2)	−0.053 (4)
C20	0.088 (5)	0.105 (10)	0.103 (8)	−0.031 (7)	−0.025 (3)	−0.029 (5)
C19	0.113 (3)	0.114 (6)	0.056 (3)	−0.046 (4)	−0.028 (3)	−0.018 (3)
C19'	0.113 (3)	0.114 (6)	0.056 (3)	−0.046 (4)	−0.028 (3)	−0.018 (3)
C20'	0.088 (5)	0.105 (10)	0.103 (8)	−0.031 (7)	−0.025 (3)	−0.029 (5)

### Geometric parameters (Å, °)

**Table:**

C1—C2	1.389 (2)	O1—S1	1.4203 (15)
C1—C6	1.392 (2)	O2—S1	1.4204 (14)
C1—N1	1.419 (2)	O3—C17	1.396 (3)
C2—C3	1.378 (3)	O3—C17'	1.478 (8)
C2—H2	0.9300	O3—P1	1.5696 (19)
C3—C4	1.392 (3)	O4—C19	1.430 (7)
C3—H3	0.9300	O4—C19'	1.444 (7)
C4—C5	1.372 (3)	O4—P1	1.5506 (17)
C4—H4	0.9300	O5—P1	1.4573 (16)
C5—C6	1.395 (2)	C17—C18	1.475 (6)
C5—H5	0.9300	C17—H17A	0.9700
C6—C7	1.434 (2)	C17—H17B	0.9700
C7—C8	1.363 (3)	C18—H18A	0.9600
C7—C16	1.428 (3)	C18—H18B	0.9600
C8—N1	1.405 (2)	C18—H18C	0.9600
C8—C15	1.488 (2)	C17'—C18'	1.492 (9)
C9—C10	1.385 (2)	C17'—H17C	0.9700
C9—C14	1.385 (2)	C17'—H17D	0.9700
C9—S1	1.7556 (17)	C18'—H18D	0.9600
C10—C11	1.383 (3)	C18'—H18E	0.9600
C10—H10	0.9300	C18'—H18F	0.9600
C11—C12	1.376 (3)	C20—C19	1.521 (9)
C11—H11	0.9300	C20—H20A	0.9600
C12—C13	1.375 (3)	C20—H20B	0.9600
C12—H12	0.9300	C20—H20C	0.9600
C13—C14	1.384 (3)	C19—H19A	0.9700
C13—H13	0.9300	C19—H19B	0.9700
C14—H14	0.9300	C19'—C20'	1.515 (9)
C15—P1	1.804 (2)	C19'—H19C	0.9700
C15—H15A	0.9700	C19'—H19D	0.9700
C15—H15B	0.9700	C20'—H20D	0.9600
C16—N2	1.134 (3)	C20'—H20E	0.9600
N1—S1	1.6851 (15)	C20'—H20F	0.9600
			
C2—C1—C6	121.35 (15)	O5—P1—O3	115.78 (10)
C2—C1—N1	131.13 (15)	O4—P1—O3	103.59 (11)
C6—C1—N1	107.51 (14)	O5—P1—C15	114.48 (10)
C3—C2—C1	117.49 (17)	O4—P1—C15	104.97 (9)
C3—C2—H2	121.3	O3—P1—C15	100.51 (10)
C1—C2—H2	121.3	O1—S1—O2	120.66 (9)
C2—C3—C4	121.69 (18)	O1—S1—N1	105.36 (8)
C2—C3—H3	119.2	O2—S1—N1	106.99 (8)
C4—C3—H3	119.2	O1—S1—C9	109.21 (8)
C5—C4—C3	120.69 (17)	O2—S1—C9	108.82 (8)
C5—C4—H4	119.7	N1—S1—C9	104.60 (7)
C3—C4—H4	119.7	O3—C17—C18	114.3 (3)
C4—C5—C6	118.56 (17)	O3—C17—H17A	108.7
C4—C5—H5	120.7	C18—C17—H17A	108.7
C6—C5—H5	120.7	O3—C17—H17B	108.7
C1—C6—C5	120.20 (16)	C18—C17—H17B	108.7
C1—C6—C7	106.83 (15)	H17A—C17—H17B	107.6
C5—C6—C7	132.95 (16)	C17—C18—H18A	109.5
C8—C7—C16	125.87 (17)	C17—C18—H18B	109.5
C8—C7—C6	109.50 (15)	H18A—C18—H18B	109.5
C16—C7—C6	124.63 (17)	C17—C18—H18C	109.5
C7—C8—N1	107.61 (15)	H18A—C18—H18C	109.5
C7—C8—C15	126.79 (16)	H18B—C18—H18C	109.5
N1—C8—C15	125.36 (16)	O3—C17'—C18'	102.5 (9)
C10—C9—C14	121.57 (15)	O3—C17'—H17C	111.3
C10—C9—S1	118.43 (12)	C18'—C17'—H17C	111.3
C14—C9—S1	119.97 (13)	O3—C17'—H17D	111.3
C11—C10—C9	118.44 (16)	C18'—C17'—H17D	111.3
C11—C10—H10	120.8	H17C—C17'—H17D	109.2
C9—C10—H10	120.8	C17'—C18'—H18D	109.5
C12—C11—C10	120.57 (18)	C17'—C18'—H18E	109.5
C12—C11—H11	119.7	H18D—C18'—H18E	109.5
C10—C11—H11	119.7	C17'—C18'—H18F	109.5
C13—C12—C11	120.47 (17)	H18D—C18'—H18F	109.5
C13—C12—H12	119.8	H18E—C18'—H18F	109.5
C11—C12—H12	119.8	C19—C20—H20A	109.5
C12—C13—C14	120.18 (17)	C19—C20—H20B	109.5
C12—C13—H13	119.9	H20A—C20—H20B	109.5
C14—C13—H13	119.9	C19—C20—H20C	109.5
C13—C14—C9	118.76 (17)	H20A—C20—H20C	109.5
C13—C14—H14	120.6	H20B—C20—H20C	109.5
C9—C14—H14	120.6	O4—C19—C20	110.0 (15)
C8—C15—P1	113.12 (12)	O4—C19—H19A	109.7
C8—C15—H15A	109.0	C20—C19—H19A	109.7
P1—C15—H15A	109.0	O4—C19—H19B	109.7
C8—C15—H15B	109.0	C20—C19—H19B	109.7
P1—C15—H15B	109.0	H19A—C19—H19B	108.2
H15A—C15—H15B	107.8	O4—C19'—C20'	106.7 (13)
N2—C16—C7	177.1 (3)	O4—C19'—H19C	110.4
C8—N1—C1	108.54 (13)	C20'—C19'—H19C	110.4
C8—N1—S1	127.93 (12)	O4—C19'—H19D	110.4
C1—N1—S1	122.66 (11)	C20'—C19'—H19D	110.4
C17—O3—C17'	61.5 (4)	H19C—C19'—H19D	108.6
C17—O3—P1	125.4 (2)	C19'—C20'—H20D	109.5
C17'—O3—P1	128.4 (4)	C19'—C20'—H20E	109.5
C19—O4—C19'	25.7 (4)	H20D—C20'—H20E	109.5
C19—O4—P1	127.1 (5)	C19'—C20'—H20F	109.5
C19'—O4—P1	122.9 (4)	H20D—C20'—H20F	109.5
O5—P1—O4	115.69 (10)	H20E—C20'—H20F	109.5
			
C6—C1—C2—C3	0.0 (2)	C2—C1—N1—S1	9.9 (2)
N1—C1—C2—C3	179.38 (16)	C6—C1—N1—S1	−170.65 (11)
C1—C2—C3—C4	−0.6 (3)	C19—O4—P1—O5	26.8 (7)
C2—C3—C4—C5	0.3 (3)	C19'—O4—P1—O5	−4.2 (5)
C3—C4—C5—C6	0.5 (3)	C19—O4—P1—O3	−100.9 (7)
C2—C1—C6—C5	0.8 (2)	C19'—O4—P1—O3	−132.0 (5)
N1—C1—C6—C5	−178.67 (14)	C19—O4—P1—C15	154.1 (7)
C2—C1—C6—C7	179.62 (15)	C19'—O4—P1—C15	123.0 (5)
N1—C1—C6—C7	0.10 (17)	C17—O3—P1—O5	−40.6 (3)
C4—C5—C6—C1	−1.1 (2)	C17'—O3—P1—O5	38.8 (6)
C4—C5—C6—C7	−179.47 (17)	C17—O3—P1—O4	87.1 (3)
C1—C6—C7—C8	0.37 (18)	C17'—O3—P1—O4	166.5 (5)
C5—C6—C7—C8	178.91 (17)	C17—O3—P1—C15	−164.5 (2)
C1—C6—C7—C16	−179.50 (16)	C17'—O3—P1—C15	−85.1 (6)
C5—C6—C7—C16	−1.0 (3)	C8—C15—P1—O5	162.95 (14)
C16—C7—C8—N1	179.19 (16)	C8—C15—P1—O4	35.01 (16)
C6—C7—C8—N1	−0.68 (18)	C8—C15—P1—O3	−72.26 (16)
C16—C7—C8—C15	4.6 (3)	C8—N1—S1—O1	149.41 (14)
C6—C7—C8—C15	−175.22 (15)	C1—N1—S1—O1	−42.48 (14)
C14—C9—C10—C11	0.2 (2)	C8—N1—S1—O2	19.85 (16)
S1—C9—C10—C11	177.98 (12)	C1—N1—S1—O2	−172.04 (12)
C9—C10—C11—C12	0.9 (3)	C8—N1—S1—C9	−95.50 (15)
C10—C11—C12—C13	−0.8 (3)	C1—N1—S1—C9	72.62 (14)
C11—C12—C13—C14	−0.2 (3)	C10—C9—S1—O1	−158.84 (12)
C12—C13—C14—C9	1.2 (3)	C14—C9—S1—O1	19.01 (15)
C10—C9—C14—C13	−1.2 (2)	C10—C9—S1—O2	−25.28 (15)
S1—C9—C14—C13	−178.99 (13)	C14—C9—S1—O2	152.57 (13)
C7—C8—C15—P1	88.3 (2)	C10—C9—S1—N1	88.79 (14)
N1—C8—C15—P1	−85.35 (18)	C14—C9—S1—N1	−93.36 (14)
C8—C7—C16—N2	167 (5)	C17'—O3—C17—C18	3.9 (6)
C6—C7—C16—N2	−13 (5)	P1—O3—C17—C18	122.6 (4)
C7—C8—N1—C1	0.74 (17)	C17—O3—C17'—C18'	−25.5 (9)
C15—C8—N1—C1	175.38 (14)	P1—O3—C17'—C18'	−139.8 (9)
C7—C8—N1—S1	170.20 (12)	C19'—O4—C19—C20	−65 (2)
C15—C8—N1—S1	−15.2 (2)	P1—O4—C19—C20	−155.4 (10)
C2—C1—N1—C8	−179.97 (17)	C19—O4—C19'—C20'	58 (2)
C6—C1—N1—C8	−0.51 (17)	P1—O4—C19'—C20'	166.2 (9)

### Hydrogen-bond geometry (Å, °)

**Table:**

Cg1 is the centroid of the C9/C10/C11/C12/C13/C14 ring and Cg2 is the centroid of the C1/C2/C3/C4/C5/C6 ring.

**Table:**

*D*—H···*A*	*D*—H	H···*A*	*D*···*A*	*D*—H···*A*
C10—H10···O5^i^	0.93	2.35	3.229 (3)	157
C5—H5···Cg1^ii^	0.93	2.63	3.501 (3)	157
C18—H18A···Cg2^iii^	0.96	2.99	3.874 (8)	154

Symmetry codes: (i) −*x*+1, −*y*+1, −*z*+1; (ii) −*x*+1, −*y*, −*z*+2; (iii) −*x*+1, −*y*, −*z*+1.

## References

[bb1] Allen, F. H. (1981). *Acta Cryst.* B**37**, 900–906.

[bb2] Bassindale, A. (1984). *The Third Dimension in Organic Chemistry*, ch. 1, p. 11. New York: John Wiley and Sons.

[bb3] Bernstein, J., Davis, R. E., Shimoni, L. & Chang, N.-L. (1995). *Angew. Chem. Int. Ed. Engl.* **34**, 1555–1573.

[bb4] Bruker (2004). *APEX2* and *SAINT* Bruker AXS Inc., Madison, Wisconsin, USA.

[bb5] Farrugia, L. J. (1997). *J. Appl. Cryst.* **30**, 565.

[bb6] Ho, C. Y., Haegman, W. E. & Perisco, F. (1986). *J. Med. Chem.* **29**, 118–121.

[bb7] Rajeswaran, W. G., Labroo, R. B., Cohen, L. A. & King, M. M. (1999). *J. Org. Chem.* **64**, 1369–1371.

[bb8] Sethu Sankar, K., Kannadasan, S., Velmurugan, D., Srinivasan, P. C. & Moon, J.-K. (2002). *Acta Cryst.* C**58**, o450–o454. 10.1107/s0108270102009587 12154297

[bb9] Sheldrick, G. M. (2008). *Acta Cryst.* A**64**, 112–122. 10.1107/S0108767307043930 18156677

[bb10] Spek, A. L. (2009). *Acta Cryst.* D**65**, 148–155. 10.1107/S090744490804362X PMC2631630 19171970

[bb11] Stevenson, G. I., Smith, A. L., Lewis, S. G., Nedevelil, J. G., Patel, S., Marwood, R. & Castro, J. L. (2000). *Bioorg. Med. Chem. Lett.* **10**, 2697–2704. 10.1016/s0960-894x(00)00557-6 11133071

